# Effects of Herbal Tea Residue on Growth Performance, Meat Quality, Muscle Metabolome, and Rumen Microbiota Characteristics in Finishing Steers

**DOI:** 10.3389/fmicb.2021.821293

**Published:** 2022-01-18

**Authors:** Ling Li, Xiaohong Sun, Junyi Luo, Ting Chen, Qianyun Xi, Yongliang Zhang, Jiajie Sun

**Affiliations:** Guangdong Provincial Key Laboratory of Animal Nutrition Control, Guangdong Laboratory for Lingnan Modern Agriculture, National Engineering Research Center for Breeding Swine Industry, South China Agricultural University, Guangzhou, China

**Keywords:** feed resources, herbal tea residue, muscle metabolism, microbial diversity, beef

## Abstract

Herbal tea residue (HTR) contains various medicinal and nutritional components and is a potential high-quality unconventional source of roughage. In this study, a total of 30 healthy Simmental crossbred finishing steers were equally divided into two groups: CN (fed with a basic diet) and RE (HTR partly replaced *Pennisetum purpureum*). HTR did not alter the growth performance of steers but increased the net meat rate, tenderness, and water-holding capacity and increased the moisture content and oleic acid and linoleic acid concentrations in *longissimus dorsi*. It altered muscle metabolic pathways and improved rumen fermentation by increasing the propionic acid concentration and propionic acid-to-acetic acid ratio. We studied the steers’ rumen microbial community composition and determined their correlation with the tested parameters. Certain rumen microorganisms were closely associated with muscle glucolipid metabolites and rumen NH_3_-N and volatile fatty acid levels. Our findings suggest that, as a functional roughage source, HTR improved to a certain extent the meat quality of steers by altering the rumen microbial composition and affecting the rumen fatty acid composition and muscle glucolipid metabolism.

## Introduction

Herbal tea, one of the most unique beverages, is a specialty drink developed according to the climatic characteristics of Lingnan in south China (Li et al., 2017). Herbal tea is mainly prepared by decocting Chinese herbal medicines such as herbal jelly, honeysuckle, chrysanthemum, prunella vulgaris, buzha leaf, and licorice (Zhao et al., 2013) and contains various bioactive substances such as flavonoids, organic acids, polysaccharides, alkaloids, and volatile oils (Liu et al., 2011). These substances usually have antibacterial, anti-inflammatory, antioxidant, antiviral, and immune-enhancing medicinal effects (Cao et al., 2014), because of which herbal teas are favored by people in the subtropical region of China.

Herbal tea residue (HTR) is the natural byproduct of preparing herbal tea. With the continuously increasing annual consumption of herbal tea, a large quantity of HTR is produced (Yang and Cui, 2013). To date, the main treatment methods for HTR are landfill, incineration, and stacking (Suthar and Singh, 2011), which not only waste resources but also pollute the environment (Malkoc and Nuhoglu, 2006). It has been reported that HTR can be used as an adsorbent for heavy metal ions to reduce water pollution (Ahsan et al., 2018). HTR is also a high-quality compost material, which significantly improves the ecological characteristics of soil (Iqbal Khan et al., 2015). Moreover, HTR is essentially a type of Chinese herbal residue (CHR), which still contains a variety of nutrients and functional active substances similar to the raw materials and can be used as animal feed (Abdallah et al., 2019). A previous study suggested that adding 0.5% CHRs to duck diets improved the crude protein content and water-holding capacity of duck meat (Jin-Woo et al., 2017). Supplementation of poultry diets with 3% CHRs improved the nutritional value, sensory quality, and tenderness of meat while reducing the antioxidant status (Kim et al., 2014). Moreover, Chinese herbal feed improves the immune function of dairy cows under heat stress (Shan et al., 2018) and contributes to rumen fermentation and energy metabolism of sheep (Liang et al., 2013).

Many recent reports recommend using a small quantity of CHRs as a functional feed additive. As a type of CHR, HTR is not only rich in protein but also contains crude fiber and trace elements that ruminant require (Xie et al., 2020). Ruminants require approximately 17% dietary crude fiber content (Ding et al., 2020). We therefore hypothesized that HTR could be used as an unconventional feed material to improve growth performance and meat quality in ruminant. In this paper, the HTR was added to the diet of steers to investigate its effects on growth performance, meat quality, muscle metabolome, rumen fermentation and rumen microbial diversity.

## Materials and Methods

### Animals, Experimental Design, and Treatments

This study was conducted at a large-scale steer farm in Guangxi, China. According to the principle of completely random allocation, 30 healthy Simmental crossbred steers (18 months old and approximately 480 kg per animal) were divided into two groups, namely CN group (fed with basic diet) and RE group (HTR partly replaced *Pennisetum purpureum*). Each experimental group included three replicates, and each replicate contained five animals. All steers were housed individually in an open cowshed at the same time. The basic composition and nutritional level of steer feed are shown in Table 1. The chemical composition of HTR was showed in Supplementary Table 1. In details, the HTR contained a high water content (75.10%) and the dry matter content was 24.90%. As a proportion of the dry matter content, the crude protein, crude fat, and ash contents were 13.10, 2.60, 6.69%, respectively. The acid detergent fiber and neutral detergent fiber were 39.8 and 54.3% (Zhuang et al., 2021). The experiment lasted for 67 days, including a 7-day pre-feeding period and a 60-day formal study period. During the experiment, the steers were fed regularly at 8 am and 5 pm, and water was available *ad libitum* throughout the experimental period. All animal procedures were approved by the Animal Care Committee at South China Agricultural University.

**TABLE 1:** Basic diet composition and nutrient level of finishing steers.

Item	CN	RE
**Ingredients**		
Corn (%)	23.7	23.7
Bean curd residue (%)	15.0	15.0
Pennisetum purpureum (%)	60.0	10.0
Herbal tea residue (%)	0	50.0
Premix (%)	1.00	1.00
Salt (%)	0.30	0.30
Total (%)	100	100
**Nutritional level**		
Dry matter (%)	34.6	30.7
Crude protein (%)	9.14	9.70
r Crude fat (%)	2.05	3.00
Neutral detergent fiber (%)	71.2	68.9
Acid detergent fiber (%)	25.2	27.6
Calcium (%)	0.69	0.63
Phosphorus (%)	0.22	0.30
Net energy (MJ/kg)	5.56	5.36

*The indicators were calculated on the basis of dry matter. The nutrient contents of the premix were as follows: Zinc, 70–100 mg/kg; Iron, 50–70 mg/kg; Copper, 30–45 mg/kg; Manganese, 6.25–10 mg/kg; Selenium, 0.3–0.5 mg/kg; Iodine, 0.2–1.00 mg/kg; Vitamin A, 7,000–10,000 IU/kg; Vitamin D, 40,000–90,000 IU/kg; Vitamin E, 4,000–5,000 mg/kg. Net energy was a calculated value, and others were measured values. CN, no herbal tea residues; RE, 50% HTRs replaced Pennisetum purpureum.*

### Measurements and Sampling

The average daily feed intake (ADFI) was recorded once a week, and individual steers were weighed at the beginning and end of the experiment to determine initial weight and average daily gain (ADG). On the last day of the experiment, 200 mL of rumen fluid per individual was obtained from 15 steers in each group before the morning feeding. Whole ruminal samples were collected from steers with a suction strainer (19 mm diameter; 1.5 mm mesh for its filter) and strained through four layers of cheesecloth (Gilbreath et al., 2020). One aliquot (100 mL) was used to determine volatile fatty acids (VFAs) using high-performance liquid chromatography (Actlabs, Ancaster, ON, Canada). Another aliquot (100 mL) was used to extract total genomic DNA for sequencing of rumen microorganisms.

At the end of the experiment, the feed was detained for 12 hours and weighed, and all animals were slaughtered at the same time. Then the carcass weight, eye muscle area (EMA), net meat weight (the weight of muscle and fat in the carcass after bone has been removed), dressing percentage, and net meat percentage were recorded for each animal post slaughter. Dressing percentage is the ratio of carcass weight to live weight at slaughter. Net meat percentage refers to net meat weight as a percentage of carcass weight. After the carcasses were chilled for 45 min at 4°C, the meat color were measured. Following aging for 24 h at 4°C, longissimus dorsi (LD) samples were collected between the 9th and 13th ribs from the right side of the carcasses, of which one was stored at 4°C for subsequent physical analysis, and the other one was frozen for nutrient value analysis.

### Meat Quality and Nutritional Composition

The meat color [lightness (L*), redness (a*), and yellowness (b*m)], water-holding capacity (drip loss and cooking loss) and tenderness (shear force) were analyzed. In detail, meat color (average of three randomly selected areas on the sample) was assessed using a Minolta Chroma Meter (CR-300, Dietikon, Switzerland), applying the L*, a*, and b* system (Razminowicz et al., 2006). Drip loss was determined as the weight loss after suspending meat samples (5 × 2 × 3 cm) at 4°C for 24 h. Meat samples in dry polyethylene bags were weighed and heated in a water bath at 85°C for 20 min and then cooled to room temperature (25°C) in running water. The cooked samples were dried and weighed again to calculate cooking loss, expressed as the percentage of uncooked sample weight (%). After measuring the cooking loss, the samples were stored for 24 h at 4°C. Subsequently, shear force was tested with a digital tenderness meter (C-LM3B, Tenovo, Beijing, China), and the average of nine replicates per sample was regarded as the final value (Sales et al., 2020). LD samples were also analyzed for moisture, dry matter, crude protein, crude fat, and ash according to Association of Official Analytical Chemists [AOAC] (2000). The fatty acid composition of frozen samples was measured by fatty acid methyl ester synthesis (O’Fallon et al., 2007). Amino acid levels were determined employing an automatic amino acid analyzer (L-8800; Hitachi, Tokyo, Japan) based on the method described by Yan et al. (2018).

### Muscle Metabolome

Non-targeted muscle metabolomics analysis was performed by Novogene Biotechnology (Beijing, China) using LC-MS platform. Specifically, approximately 100 mg of frozen samples were ground and homogenized in 500 μL of 80% methanol containing 0.1% formic acid. The mixtures were kept in an ice bath for 5 min, and then centrifuged at 15,000 × *g* for 10 min at 4°C. After the content of methanol in the supernatant was diluted to 53%, the mixture was centrifuged again (15,000 × *g* for 10 min at 4°C). Then, the supernatant (200 μL) was transferred to an LC-MS sampling vial for LC-MS analysis. Raw data were filtered and aligned by parameter (retention time, mass-to-charge ratio, and peak intensity) selection of Compound Discoverer 3.1 software (Thermo Scientific). The processed data were used to annotate the metabolites using the KEGG, HMDB and LIPID MAPS databases. Moreover, the dataset of two groups was separated with partial least squares discriminant analysis (PLS-DA). The differentially expressed metabolites between two groups are illustrated with a volcano plot.

### 16S rRNA Gene Sequencing and Annotation Analysis of Rumen Microorganisms

Total genomic DNA was extracted from rumen fluid samples using the SDS method, and the integrity of the extracted DNA was assessed by 1% agarose gel electrophoresis (Black and Foarde, 2007). DNA concentration was determined using Qubit Fluorometer (Invitrogen, Carlsbad, CA). 16S rRNA genes were subsequently amplified using specific primers with barcode (Forward: 5′-GTGCCAGCMGCCGCGG-3′ and Reverse: 5′-GGACTACHVGGGTWTCTAAT-3′) targeting the variable regions V3–V4. The sequencing library was prepared using the gDNA samples using the Illumina TruSeq^®^ DNA PCR-Free Sample Preparation Kit. Qubit and Real-Time PCR System were used to assess the quantity and quality of the sample library. Then, the library constructed was sequenced using NovaSeq6000 platform. Clean reads were obtained from the raw data by strict quality filtering and chimeric sequence removal (Haas et al., 2011). The effective tags of all samples were clustered, and the tags with over 97% similarity were regarded as one operational taxonomic unit (OTU) (Edgar, 2013). According to the Silva 132 database, a representative sequence for each OTU was screened for taxonomic identification based on the Mothur algorithm (Quast et al., 2013). To explore the phylogenetic relationship among different OTUs, multiple sequence alignment was performed using MUSCLE software (Version 3.8.31) (Yuan et al., 2018). All the data were normalized, and the least amount of data were considered as the standard. The subsequent alpha-diversity and beta-diversity analysis were based on the normalized data. Alpha-diversity analysis reflected the complexity and diversity of species for the samples, including the observed species, Simpson, Shannon, Chao1 and ACE indices. For beta-diversity analysis, principal coordinate analysis (PCoA) was performed to obtain the principal coordinates and visualize complex, multidimensional data. Non-metric multi-dimensional scaling (NMDS) was employed to visualize and compare the relationship of the rumen microbial community structure between the two groups. Unweighted pair group method with arithmetic means (UPGMA) clustering was conducted as a type of hierarchical clustering method to interpret the distance matrix using average linkage. Linear discriminant analysis effect size (LEfSe) method was employed to identify statistically significant biomarkers between groups.

### Statistical Analysis

Growth performance, carcass characteristics, meat quality, meat nutrition level, and rumen VFA content were analyzed by the independent sample *t*-test using SPSS software 17.0 (IBM Corp., Armonk, NY, United States). The correlation analyses of rumen microbiota with the tested traits were performed using the function cor (x, y, use = “p”) and illustrated with function labeledHeatmap (Matrix, xLabels, yLabels) in the R package WGCNA (Langfelder and Horvath, 2008). The data were expressed as mean ± standard error of the mean (SEM), and statistical significance was established at *P* < 0.05.

## Results

### Growth Performance and Carcass Characteristics

The growth performance and carcass characteristics between the two groups during the finishing phase are listed in Table 2. The growth performance parameters initial weight, live weight at slaughter, ADFI, and ADG did not differ between treatments (*P* > 0.05). The carcass weight, dressing percentage, net meat weight, and EMA were not significantly different between groups (*P* > 0.05), but the net meat rate was higher in the RE group than in the CN group (41.72 and 40.28%, respectively; *P* < 0.05).

**TABLE 2:** Effects of herbal tea residue feed on the growth performance and carcass characteristics of finishing steers.

Parameter	CN	RE	*P*-value
Initial weight (kg)	479.87 ± 10.99	482.53 ± 6.95	0.24
Live weight at slaughter (kg)	542.91 ± 11.68	547.09 ± 7.56	0.28
Average daily feed intake (kg)	12.64 ± 0.30	12.33 ± 0.28	0.79
Average daily gain (kg)	1.05 ± 0.04	1.08 ± 0.05	0.72
Carcass weight (kg)	307.58 ± 18.02	319.01 ± 16.53	0.65
Dressing percentage (%)	56.54 ± 0.90	57.26 ± 0.46	0.49
Net meat weight (kg)	257.58 ± 18.02	267.58 ± 15.71	0.68
Net meat percentage (%)	40.28 ± 0.38	41.72 ± 0.48	0.04
Eye muscle area (cm^2^)	78.82 ± 5.03	81.70 ± 6.09	0.72

*The values were calculated as the mean ± standard error of the mean (N = 15). P < 0.05 indicated a significant difference between the two groups; P > 0.05 indicated no significant difference between the two groups. CN, no herbal tea residues; RE, 50% HTR replaced Pennisetum purpureum.*

### Meat Quality and Nutritional Composition

The LD quality traits are presented in Table 3. Compared with the CN group, the RE group showed a significantly lower drip loss (5.85% vs. 4.45%, *P* < 0.01), cooking loss (29.96% vs. 27.58%, *P* < 0.01) and shear force (65.26 N vs. 48.13 N, *P* < 0.01). For meat color-related parameters (L*, a*, and b*), the values of a* and b* were not significantly different between treatments, but the L* value of the RE group was higher than that of CN group (34.96 and 36.91, respectively). The crude protein and crude fat contents were significantly different between groups. However, the moisture content in the CN and RE groups was 3.30 and 4.69%, respectively, indicating a significant increase (*P* < 0.01) when Simmental steers were fed with diets containing HTR. The content of crude ash was lower in the RE group than in the CN group (0.038% vs. 0.044%, *P* < 0.05; Table 4). The amino acid composition in LD was not significantly different between groups (Supplementary Table 2). Regarding fatty acid composition (Table 5), the content of oleic acid (C18:1n9c) and linoleic acid (C18:2n6t) content accounted for 37.35% and 0.11% of fatty acids in the CN group and 39.74% and 0.18% of fatty acids in the RE group, respectively; their content in the RE group was significantly higher than that in the CN group (*P* < 0.05). Moreover, the ratio of ω-6/ω-3 fatty acids in the RE group was lower than that in the CN group (*P* = 0.06).

**TABLE 3:** Effects of herbal tea residue feed on the meat quality of finishing steers.

Parameter	CN	RE	*P-*value
Drip loss (%)	5.85 ± 0.304	4.45 ± 0.233	0.001
Cooking loss (%)	29.96 ± 0.752	27.58 ± 0.443	0.006
Shear force (N)	65.26 ± 3.404	48.13 ± 2.314	0.001
Meat color			
Lightness (L*)	34.96 ± 0.662	36.91 ± 0.55	0.032
Redness (a*)	18.91 ± 0.723	18.68 ± 0.753	0.823
Yellowness (b*)	9.50 ± 0.534	9.85 ± 0.452	0.624

*The values were calculated as the mean ± standard error of the mean (N = 15). The shear force was calculated as the average of nine replicates per sample. P < 0.05 indicated a significant difference between the two groups; P > 0.05 indicated no significant difference between the two groups. CN, no herbal tea residue; RE, 50% HTR replaced Pennisetum purpureum.*

**TABLE 4:** Effects of herbal tea residue feed on the basic nutritional composition of beef.

Parameter	CN	RE	*P-*value
Moisture (%)	3.30 ± 0.18	4.69 ± 0.16	0.002
Crude protein (%)	86.19 ± 1.38	88.04 ± 1.08	0.33
Crude fat (%)	0.43 ± 0.003	0.46 ± 0.00	0.53
Crude ash (%)	0.044 ± 0.001	0.038 ± 0.002	0.04

*The indicators were calculated on the basis of lyophilized samples. The values were calculated as the mean ± standard error of the mean (N = 15). P < 0.05 indicated a significant difference between the two groups; P > 0.05 indicated no significant difference between the two groups. CN, no herbal tea residue; RE, 50% HTR replaced Pennisetum purpureum.*

**TABLE 5:** Effects of herbal tea residue feed on the fatty acid content of beef (g/100 g).

Fatty acid	CN	RE	*P-*value
Decanoic acid (C10:0)	0.07 ± 0.007	0.06 ± 0.007	0.289
Lauric acid (C12:0)	0.10 ± 0.013	0.08 ± 0.005	0.343
Myristic acid (C14:0)	2.63 ± 0.146	2.41 ± 0.150	0.332
Myristoleic acid (C14:1)	0.44 ± 0.052	0.66 ± 0.108	0.061
Pentadecanoic acid (C15:0)	0.45 ± 0.036	0.46 ± 0.052	0.830
Palmitic acid (C16:0)	26.70 ± 0.453	25.57 ± 0.516	0.123
Palmitoleic acid (C16:1)	3.44 ± 0.141	3.39 ± 0.249	0.851
Heptadecanoic acid (C17:0)	0.97 ± 0.061	1.21 ± 0.130	0.106
10-Heptadecenoic acid (C17:1)	0.65 ± 0.057	0.69 ± 0.034	0.566
Stearic acid (C18:0)	18.18 ± 0.597	17.59 ± 0.651	0.513
Oleic acid (C18:1n9c)	37.345 ± 0.536	39.74 ± 0.855	0.036
Linoleic acid (C18:2n6t)	0.11 ± 0.010	0.18 ± 0.031	0.050
Methyl linoleate (C18:2n6c)	4.27 ± 0.406	4.50 ± 0.618	0.772
α-Linolenic acid (C18:3n3)	0.40 ± 0.050	0.43 ± 0.034	0.577
Arachidic acid (C20:0)	0.15 ± 0.016	0.15 ± 0.008	0.817
11,14,17-Eicosatrienoic acid (C20:3n3)	0.59 ± 0.266	0.53 ± 0.182	0.840
Methyl arachidonic acid (C20:4n6)	1.55 ± 0.329	2.20 ± 0.794	0.454
Eicosapentaenoic acid (C20:5n3)	0.24 ± 0.068	0.25 ± 0.074	0.899
Heneicosanoic acid-methyl ester (C21:0)	0.24 ± 0.062	0.21 ± 0.038	0.696
Docosanoic acid (C22:0)	0.06 ± 0.012	0.05 ± 0.020	0.520
Methyl cis-13,16-docosadienoic acid (C22:2)	0.13 ± 0.032	0.14 ± 0.023	0.896
Nervonic acid (C24:1)	0.21 ± 0.030	0.21 ± 0.055	0.999
Saturated fatty acid (SFA)	49.57 ± 0.439	48.95 ± 0.632	0.437
Unsaturated fatty acid (UFA)	50.24 ± 0.461	51.07 ± 0.738	0.359
Omega-6 (ω-6)	7.07 ± 1.145	6.64 ± 1.373	0.811
Omega-3 (ω-3)	1.18 ± 0.360	1.27 ± 0.302	0.846
ω-6: ω-3	6.98 ± 0.358	5.99 ± 0.334	0.060

*The values were calculated as the mean ± standard error of the mean (N = 15). P < 0.05 indicated a significant difference between the two groups; P > 0.05 indicated no significant difference between the two groups. CN, no herbal tea residue; RE, 50% HTR replaced Pennisetum purpureum.*

### Muscle Metabolome

**FIGURE 1:**
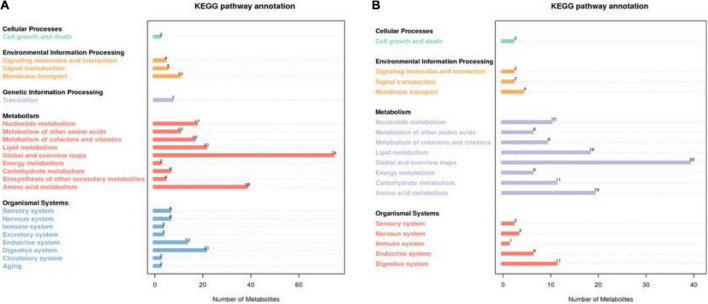
Differential metabolite annotation statistics in the KEGG database in the positive ion mode **(A)** and the negative ion mode **(B)**. The X-axis represents the number of metabolites, and the y-axis represents the KEGG term.

**FIGURE 2:**
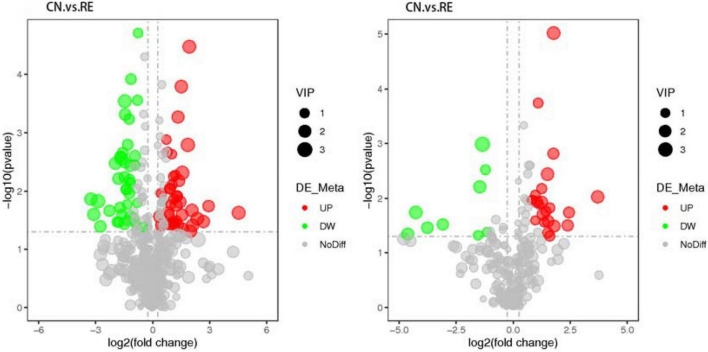
Volcanic map of differential metabolites (positive ion mode on the left and negative ion mode on the right). The X-axis represents the expression multiple change (log2FoldChange) of metabolites in different groups; and the Y-axis represents the significance level of difference (–log10p-value). Each point in the volcanic map represents a metabolite; the significantly upregulated metabolites are represented by red dots, whereas the significantly downregulated metabolites are represented by green dots.

A total of 774 metabolites were detected in the steer muscles, including 519 in the positive ionization mode and 255 in the negative ionization mode (Supplementary Tables 3A,B). The annotation results obtained using biological databases (KEGG, HMDB, LIPID MAPS) suggest that these metabolites were mainly involved in the metabolic pathways of lipid metabolism, carbohydrate metabolism, and amino acid metabolism (Figure 1 and Supplementary Figure 1). A total of 21, 6, and 38 metabolites in the positive ion mode were mainly involved in lipid, carbohydrate, and amino acid metabolism pathways, whereas 18, 11, and 19 metabolites in the negative ion mode were associated with lipid, carbohydrate, and amino acid metabolism (Supplementary Tables 3C,D). As shown in Supplementary Figure 2A (R2Y = 0.84, Q2Y = 0.56) and 2B (R2Y = 0.78, Q2Y = 0.39), the PLS-DA model revealed a clear separation between muscle metabolomes of steers fed with different diets. Supplementary Tables 3E,F summarize all the differential metabolites of the two groups in the positive and negative ion mode, as well as their query IDs, *P*-value, and fold change (FC). Based on FC threshold ≥ 2 (or ≤ 0.5) and a *P*-value < 0.05; 90 metabolites, including 30 upregulated and 32 downregulated metabolites in the positive ion mode and 19 upregulated and 9 downregulated metabolites in the negative ion mode, were significantly altered between the two groups (Figure 2). Moreover, we found that many differential metabolites were associated with glucose and lipid metabolism pathways. Phosphocholine, linolenic acid, and D-glucose 6-phosphate (G6p) showed significantly lower levels in the muscle in RE animals than in CN animals, whereas adenosine 5′-monophosphate (AMP), androstenedione, arachidonic acid (ARA), caprylic acid, cortisol, cortisone, docosahexaenoic acid (DHA), docosapentaenoic acid (DPA), D-glucarate, histamine, lauric acid, and progesterone showed significantly higher levels in RE animals (*P* < 0.05).

### NH_3_-N and Volatile Fatty Acid Concentrations in Rumen Fluid

The rumen NH3-N and VFA concentrations are listed in Table 6. The concentration of propionic acid in the CN and RE groups was 8.40 and 10.38 mmol/L, respectively, with the RE group showing significantly higher levels than the CN group (*P* < 0.05). The ratio of propionic acid to acetic acid levels in the RE group was also significantly higher than that in the CN group (0.22 and 0.24, respectively; *P* < 0.05). However, there were no significant differences between the CN and RE groups in the concentrations of acetic acid, isobutyric acid, butyric acid, isovaleric acid, and valeric acid (*P* > 0.05).

**TABLE 6:** Effects of herbal tea residue feed on the rumen fermentation parameters of finishing steers.

Metabolite	CN	RE	*P-*value
Ammonia-N (mg/100 mL)	8.80 ± 1.053	11.43 ± 2.435	0.396
Acetic acid (mmol/L)	35.88 ± 2.660	30.58 ± 3.385	0.233
Propionic acid (mmol/L)	8.40 ± 0.343	10.38 ± 0.659	0.019
Isobutyric acid (mmol/L)	0.69 ± 0.109	0.59 ± 0.085	0.457
Butyric acid (mmol/L)	3.89 ± 0.299	3.92 ± 0.436	0.956
Isovaleric acid (mmol/L)	0.74 ± 0.145	0.64 ± 0.075	0.512
Valeric acid (mmol/L)	0.24 ± 0.043	0.19 ± 0.022	0.325
Propionic acid/Acetic acid	0.22 ± 0.007	0.24 ± 0.006	0.023

*The values were calculated as the mean ± standard error of the mean (N = 15). P < 0.05 indicated a significant difference between the two groups; P > 0.05 indicated no significant difference between the two groups. CN, no herbal tea residue; RE, 50% HTR replaced Pennisetum purpureum.*

### 16S rRNA Gene Sequencing and Annotation Analysis

**FIGURE 3:**
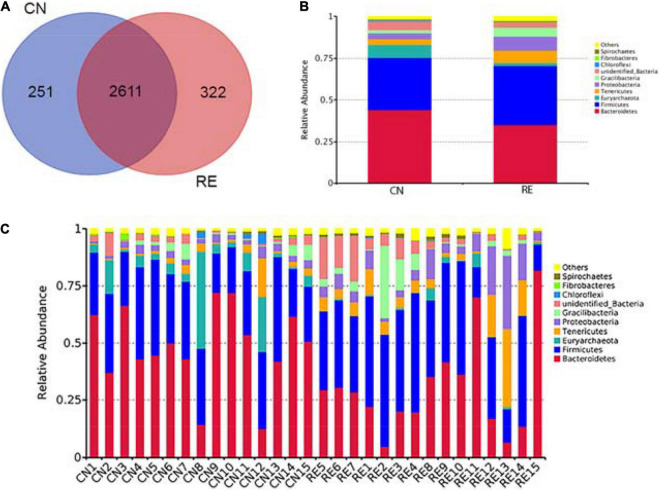
Venn diagram representing the shared and exclusive OTUs at the 97% similarity level between rumen microbiota in the two groups **(A)**. Bar plot shows the relative abundance of rumen microbiota at the phylum level in each group **(B)** and in each sample **(C)**. OTUs, operational taxonomic units; CN, no herbal tea residue (control); RE, 50% HTR replaced *Pennisetum purpureum*.

The high-throughput sequencing analysis generated a total of 2,442,873 raw reads. On average, each sample yielded approximately 81,429 joined tags, and more than 61.09% of the total joined tags in each sample were processed for subsequent analysis after data filtering, quality control, and chimera removal (Supplementary Table 4A). A total of 3,184 OTUs were identified on the basis of 97% nucleotide sequence similarity; of these, 2,611 OTUs were found across all groups and defined as core OTUs. The number of unique OTUs in the CN and RE groups was 251 and 322, respectively (Figure 3A and Supplementary Table 4B). We annotated all these OTU tags to the Silva132 database and found that 92.09% of the sequences were assigned at the phylum level, whereas 89.42, 81.88, 71.39, 30.59, and 10.27% of the annotated OTUs were assigned at the class, order, family, genus, and species levels, respectively (Supplementary Table 4C). Phylogenetic analysis identified the top 10 phyla from the rumen of steers using the QIIME pipeline on the basis of the OTU annotation (Figures 3B,C). The dominant phyla in the rumen of steers were Bacteroidetes, Firmicutes, and Euryarchaeota, accounting for 43.95, 31.45, and 7.76%, respectively. And the Firmicutes to Bacteroidetes ratio increased from 0.72 in the CN group to 1.02 in the RE group (Supplementary Table 5A). At the genus level, a total of 301 classifiable genera were detected, and nine genera had a relative abundance greater than 1.0%, including *Methanobrevibacter, Anaeroplasma, Bacteroidales, Mycoplasma, Candidatus Saccharimonas, Lachnospiraceae, Ruminococcaceae, Prevotellaceae*, and *Saccharofermentans*. The most abundant genus in the rumen of Simmental steers was *Methanobrevibacter* (4.59%) (Supplementary Table 5B).

### Changes in Rumen Microorganisms

**FIGURE 4:**
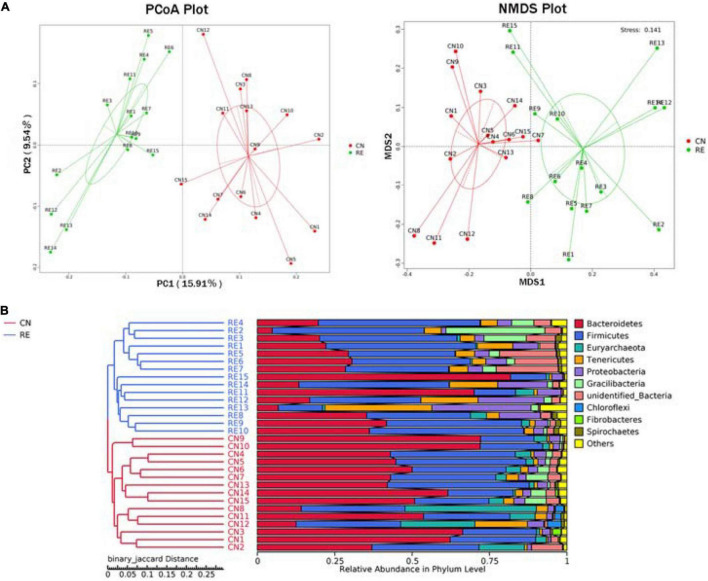
**(A)** Principal coordinate analysis (PCoA) and non-metric multi-dimensional scaling (NMDS). **(B)** Unweighted pair group method with arithmetic means (UPGMA) cluster tree based on binary Jaccard distance. **(A)** The PCoA was based on the unweighted UniFrac distance, and the NMDS analysis was based on the Bray–Curtis distance. Each point in the figure represents a sample, and the samples in the same group are represented by the same color. **(B)** On the left is the UPGMA cluster tree structure of each sample at the OUT level, and on the right is the relative abundance distribution map of each sample at the phylum level. CN, no herbal tea residues; RE, 50% HTR replaced *Pennisetum purpureum*.

**FIGURE 5:**
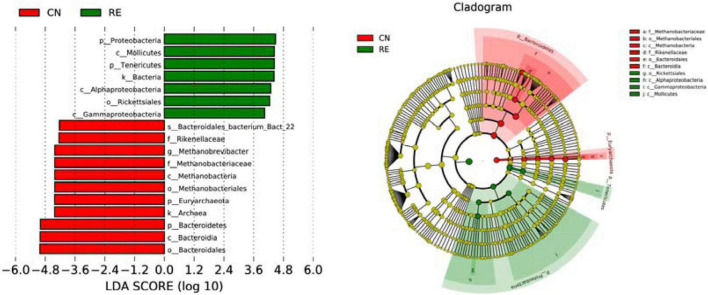
Comparison of the classification of rumen microbiota between two groups by linear discriminant analysis effect size (LefSe) method. The LDA value distribution histogram (left) shows the species with significant differences in abundance in the two groups, and the length of the histogram represents the impact of different species. In the taxonomic cladogram (right), the circles radiating from the inside to the outside represent the classification level from phylum to species. Taxa with enriched levels in CN are shown in red, whereas those with enriched levels in RE are shown in green. The species names indicated by the letters in the picture are shown in the legend on the right.

For the alpha-diversity analysis, we calculated the observed species index, Shannon, Simpson, Chao1, ACE, Good’s coverage, PD whole tree indices for each group. Although alpha diversity in RE groups tended to decrease compared with the control, these differences did not significantly affect species level microbial diversity (*P* > 0.05; Table 7). The results of the PCoA and NMDS analysis between the groups are shown in Figure 4A. The CN and RE samples were separated from each other, which reflects the effect of HTR on the rumen microbial community. The UPGMA clustering tree (Figure 4B) measured the similarity in microbial communities between groups according to the degree of their overlap and confirmed the significant structural separation of the rumen microflora between the two groups. The LEfSe analysis was used to identify the biomarkers between the two groups, and 18 differentially abundant taxonomic clades were found, with a Linear Discriminant Analysis (LDA) score higher than 4. The number of biomarkers at the kingdom, phylum, class, order, family, genus and species levels were 2, 4, 5, 3, 2, 1, and 1 respectively (Figure 5, left). A total of 11 taxa can be used as biomarkers for CN samples, including Archaea at the kingdom level, Bacteroidetes and Euryarchaeota at the phylum level, Bacteroidia and Methanobacteriales at the class level, Bacteroidales and Methanobacteriales at the order level, *Methanobacteriaceae* and *Rikenellaceae* at the family level, *Methanobrevibacter* at the genus level, and *Bacteroidales-bacterium-Bact-22* at the species level. A total of seven taxa can be used as biomarkers for RE samples, including Bacteria at the kingdom level; Proteobacteria and Tenericutes at the phylum level; Mollicutes, Alphaproteobacteria, and Gammaproteobacteria at the class level, and Rickettsiales at the order level (Figure 5, right). A total of 23 genera displayed a significant difference in abundance between the CN and RE groups, including 3 upregulated and 20 downregulated genera (*FDR* < 0.05). Specifically, the abundance of *Riemerella* in the CN group and the abundance of *Rikenellaceae*, *Anaerovorax*, *Desulfovibrio*, *Papillibacter*, *Succiniclasticum*, *Veillonellaceae*, *Acetitomaculum*, *Christensenellaceae*, and *Schwartzia* in the RE group was significantly decreased (*FDR* < 0.01; Supplementary Table 5C).

**TABLE 7:** Effects of herbal tea residue feed on the alpha diversity indices for bacteria in the ruminal samples of finishing steers.

Items	CN	RE	*P*-value
Observed species	1179.60 ± 50.59	1100.47 ± 56.37	0.305
Shannon	6.81 ± 0.28	6.61 ± 0.28	0.634
Simpson	0.95 ± 0.01	0.95 ± 0.01	0.947
Chao1	1447.77 ± 110.81	1332.69 ± 68.89	0.385
ACE	1426.82 ± 61.86	1353.86 ± 64.65	0.422
Good’s coverage	0.99 ± 0.00	0.99 ± 0.00	0.711
PD whole tree	88.93 ± 3.96	87.41 ± 2.90	0.759

*The values were calculated as the mean ± standard error of the mean (N = 15). P < 0.05 indicated a significant difference between the two groups, and P > 0.05 indicated no significant difference between the two groups. CN, no herbal tea residue; RE, 50% HTR replaced Pennisetum purpureum.*

### Correlation of Rumen Microbiota Abundance With Volatile Fatty Acid and NH3-N Concentration

Pearson correlation analysis was performed to further identify the relationship between the relative abundance of differential bacterial genera identified by 16S rRNA sequencing with rumen VFA and NH3-N concentration (Supplementary Figure 3). The concentration of acetic acid, propionic acid, and butyric acid correlated negatively with the relative abundance of *Riemerella* (*P* = 0.01, *P* = 0.03, and *P* = 0.03, respectively) and Moraxella (*P* = 0.01, *P* = 0.03, and *P* = 0.04, respectively). The concentration of propionic acid was negatively correlated with the relative abundance of *Marvinbryantia* (*P* = 0.03). The concentration of isovaleric acid, valeric acid, and NH_3_-N showed a significant positive correlation with the relative abundance of *Veillonellaceae* (*P* = 0.03, *P* = 0.05, and *P* = 0.02, respectively), *Olsenella* (*P* = 0.008, *P* = 0.01, and *P* = 0.009, respectively) and *Schwartzia* (*P* = 0.01, *P* = 0.002, and *P* = 0.02, respectively). Additionally, the concentration of isobutyric acid was most highly correlated with the relative abundance of *Olsenella* (*P* = 0.002).

### Correlation Between Rumen Microbiota and Muscle Glycolipid Metabolites

**FIGURE 6:**
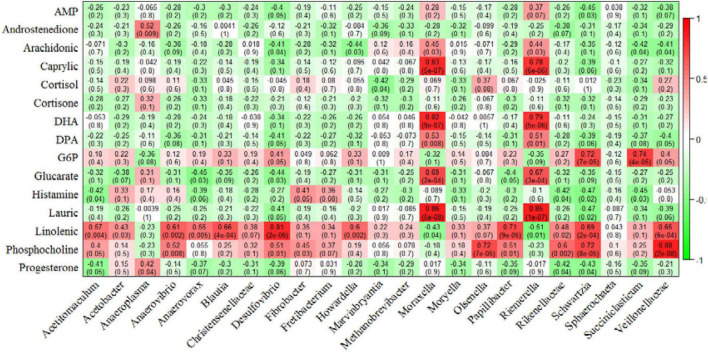
Correlation analysis of rumen microorganisms with beef glycolipid metabolite concentrations. Each cell contains the corresponding correlation and *P*-value. The table is color-coded by correlation according to the color legend. AMP, adenosine monophosphate; DHA, docosahexaenoic acid; DPA, docosapentaenoic acid; G6P, glucose-6-phosphate.

We also performed a correlation analysis between differential rumen microorganisms with muscle differential glucolipid metabolites (Figure 6). The concentration of caprylic acid, DHA, DPA, glucarate, and lauric acid displayed a strong and positive correlation with relative abundance of *Moraxella* and *Riemerella*, respectively (*P* < 0.01). The concentration of linolenic acid showed a positive correlation with the relative abundance of *Acetitomaculum*, *Anaerovibrio*, *Anaerovorax*, *Blautia*, *Desulfovibrio*, *Howardella*, *Papillibacter*, *Schwartzia*, *Veillonellaceae* (*P* < 0.01) and a negative correlation with the relative abundance of *Riemerella* (*P* = 0.01). The concentration of phosphocholine was positively related to the bacterial abundance of *Anaerovibrio*, *Desulfovibrio*, *Olsenella*, *Papillibacter*, *Rikenellaceae*, *Schwartzia*, and *Veillonellaceae* (*P* < 0.01). In addition, the concentration of G6P correlated strongly and positively with the relative abundance of *Schwartzia* and *Succiniclasticum* (*P* < 0.01).

## Discussion

HTR still retain a considerable proportion of the nutrients and bioactive compounds, which has the potential to be used as an unconventional feed resource for ruminant (Xie et al., 2020). The results of the study showed that the HTR as a part of the diet has no adverse effects on the growth performance of finishing steers, and this is in accordance with our early reports of Zhuang et al. (2021). In this study, HTR had no significant effect on carcass weight, dressing percentage and EMA of finishing steers, but significantly improved the net meat percentage. This could be the ample nutrients and active ingredients in HTR to improve the fattening degree of finishing steers (Brscic et al., 2014). Meat quality is an important economic trait of bovine husbandry. Tenderness, water-holding capacity, and color are the vital but highly variable attributes of beef quality (Modzelewska-Kapituła et al., 2018). In the present study, the meat drip loss, cooking loss, and shear force were significantly lower in the RE group than in the CN group. This finding is consistent with the findings reported by Ding et al. (2020), who showed that tea residues increased moisture content and tenderness in pork. The increase in the L * value in RE group was associated with the high moisture content of beef (Barahona et al., 2016). According to our findings, HTR could improve the meat quality of finishing steers to a certain extent.

Regarding the muscle nutrient composition, the content of C18:1n9c and C18:2n6t was higher, and the ω-6/ω-3 ratio was slightly lower when the steers were fed with HTR. C18:1n9c, a monounsaturated fatty acid, can regulate blood lipids and lowers cholesterol (Sales-Campos et al., 2013). C18:2n6t, a type of *trans*-fatty acid produced by ruminants, has a potential protective effect against the development of coronary heart diseases (Salter, 2013). Moreover, a low ω-6/ω-3 ratio in beef is more beneficial for human health as it decreases the risk of heart disease and cancer (Kang, 2004). Ahmed et al. (2016) reported that Chinese herbal medicine feed additive improved the nutritional value of pork by decreasing the ω-6/ω-3 value. Thus, the findings suggest that the beef of the experimental group is more appropriate for human diet.

In this study, HTR did not affect within-sample diversity (species richness and evenness), however, the composition and structure of the rumen microbial community were influenced by HTR. In steers fed with HTR, the abundance of *Bacteroidetes* markedly decreased, whereas the abundance of *Firmicute*s markedly increased, and it became the most abundant phylum. In humans and mice, an increase in the *Firmicutes*-to-*Bacteroidetes* ratio has been correlated with fat deposition in tissues (Ley et al., 2006; Turnbaugh et al., 2006). In our study, the crude fat content of muscle did show an upward trend with an increasing Firmicutes-to-Bacteroidetes ratio. The abundance of the genera *Veillonellaceae*, *Schwartzia*, and *Olsenella* decreased in the rumen of Simmental steers fed with HTR. Notably, these genera showed a strong positive correlation with isobutyric acid, valeric acid, and isovaleric acid concentrations. Compared with CN group, isobutyric acid, valeric acid, and isovaleric acid concentrations presented a decreasing trend in the RE group. This finding is consistent with previous findings that these bacteria were positively correlated with rumen VFAs (Kong et al., 2019; Li et al., 2019; Wang et al., 2021). Genus *Marvinbryantia* was also downregulated in the RE group compared with the CN group. The relative abundance of *Marvinbryantia* correlated negatively with the concentrations of propionic acid. Wang et al. (2018) reported that *Marvinbryantia* was an inflammatory bacterium and was negatively correlated with VFA concentration. These findings suggest that HTR reduces the proliferation of inflammatory bacteria. An increase in propionic acid and propionic acid-to-acetic acid ratio was observed in this study, which is often observed as a result of enhanced digestion of fiber and the proliferation of microorganisms in the rumen and may induce changes in metabolic pathways and better rumen fermentation (Christensen et al., 2015). This finding is in agreement with the findings reported by Zhu et al. (2018) and Liang et al. (2019), who suggested that Chinese herbal mixture feed additives improved rumen fermentation, mainly by increasing the concentration of propionic acid and the ratio of propionic acid to acetic acid. Notably, propionate is the main source of glucose for ruminants (den Besten et al., 2013), which may explain the improved carcass characteristics, especially net meat percentage, observed in the RE group in the present study.

Metabolomics analysis showed that the levels of D-glucarate, caprylic acid, lauric acid, DHA, and DPA were higher in the RE group. D-glucarate is oxidized to D-glycerate by glucose oxidase. D-glycerate is a crucial component of pentose phosphate pathway (PPP), which is involved in the first step of glucose metabolism in the glycolysis branch (Stincone et al., 2015). Medium-chain fatty acids (e.g., caprylic acid and lauric acid) are known to contribute to better beef flavor and odor and improve cholesterol levels (Wilson et al., 2006). DHA and DPA are the most bioactive of ω-3 polyunsaturated fatty acids (PUFAs) and play vital roles in decreasing the hepatic triglyceride content (Pirillo and Catapano, 2013), and PUFAs are known to be beneficial for human health (Russo, 2009). This is consistent with our results of the fatty acid analysis, which showed a reduction in the ω-6: ω-3 ratio. Remarkably, the abundance of *Moraxella* and *Riemerella* increased in the rumen of Simmental steers fed HTR, and the abundance of these bacteria was positively correlated with the levels of D-glucarate, caprylic acid, lauric acid, DHA, and DPA. Currently, there is no information on specific associations between these bacteria and the above muscle metabolites. In addition, the concentration of G6P correlated strongly and positively with the relative abundance of *Succiniclasticum*, which includes starch-degrading bacteria that degrade dietary starch (Fernando et al., 2010; Huws et al., 2016). The abundance of *Anaerovibrio* and *Papillibacter* correlated positively with the concentration of linolenic acid. *Anaerovibrio* participates in the breakdown of fats and sugars (Ouattara et al., 1992; Mannelli et al., 2018). *Papillibacter* belongs to the *Ruminococcaceae* family; members of the *Ruminococcaceae* family are essential for cellulose degradation (Krause et al., 2003). The specific association of *Anaerovibrio* and *Papillibacter* with linolenic acid remains unclear and requires an in-depth investigation in the future. The present findings suggest that HTR improves muscle glucolipid metabolism and rumen fermentation by altering the microbial community composition. However, more systematic studies should be included to reveal the biological associations.

## Conclusion

The present study showed that HTR improved meat quality to a certain extent, influenced the muscle metabolic pathways, and altered the rumen VFA concentration and rumen microbial community composition in Simmental crossbred finishing steers. Moreover, the bacteria were closely associated with muscle glucolipid metabolites and rumen VFA levels of the steers. Our findings suggest that, as a functional roughage, HTR improves the meat quality of steers mainly by altering rumen microbial populations and then affecting rumen fatty acid composition and muscle glucolipid metabolism.

## Data Availability Statement

The original contributions presented in the study are included in the article/Supplementary Material, further inquiries can be directed to the corresponding author/s.

## Ethics Statement

The animal study was reviewed and approved by the Animal Care Committee at South China Agricultural University.

## Author Contributions

LL: writing-original draft preparation. XS: investigation. JS: data curation and visualization. JL, TC, and QX: supervision. YZ and JS: conceptualization, methodology, writing-reviewing, and editing. All authors contributed to the article and approved the submitted version.

## Conflict of Interest

The authors declare that the research was conducted in the absence of any commercial or financial relationships that could be construed as a potential conflict of interest.

## Publisher’s Note

All claims expressed in this article are solely those of the authors and do not necessarily represent those of their affiliated organizations, or those of the publisher, the editors and the reviewers. Any product that may be evaluated in this article, or claim that may be made by its manufacturer, is not guaranteed or endorsed by the publisher.

## Supplementary Material

Supplementary Figure 1 Differential metabolites annotation statistics in HMDB **(A)** and LIPID MAPS **(B)** database. The left picture is the positive ion mode, and the right picture is the negative ion mode. The X-axis represents the number of metabolites, and the y-axis represents the term of HMDB or LIPID MAPS.

Supplementary Figure 2 The PLS-DA scatter plots of each sample in positive (left) and negative (right) modes. The abscissa is the score of the sample on the first principal component; The ordinate is the score of the sample on the second principal component; R2Y represents the explanatory rate of the model, and Q2Y is used to evaluate the predictive ability of the PLS-DA model. When R2Y is greater than Q2Y, the model is well established.

Supplementary Figure 3 Correlation analysis of rumen microorganisms with rumen volatile fatty acid concentrations. Each cell contains the corresponding correlation and *P*-value. The table is color-coded by correlation according to the color legend.
