# Supplementary material for: Effects of Herbal Tea Residue on Growth Performance, Meat Quality, Muscle Metabolome, and Rumen Microbiota Characteristics in Finishing Steers
Source: Front Microbiol. 2022 Jan 18;12:821293. doi: 10.3389/fmicb.2021.821293 (PMC8804378; doi:10.3389/fmicb.2021.821293)
Supplement: Supplementary Figure 1 — Differential metabolites annotation statistics in HMDB (A) and LIPID MAPS (B) database. The left picture is the positive ion mode, and the right picture is the negative ion mode. The X-axis represents the number of metabolites, and the y-axis represents the term of HMDB or LIPID MAPS. [file Image_1.pdf]

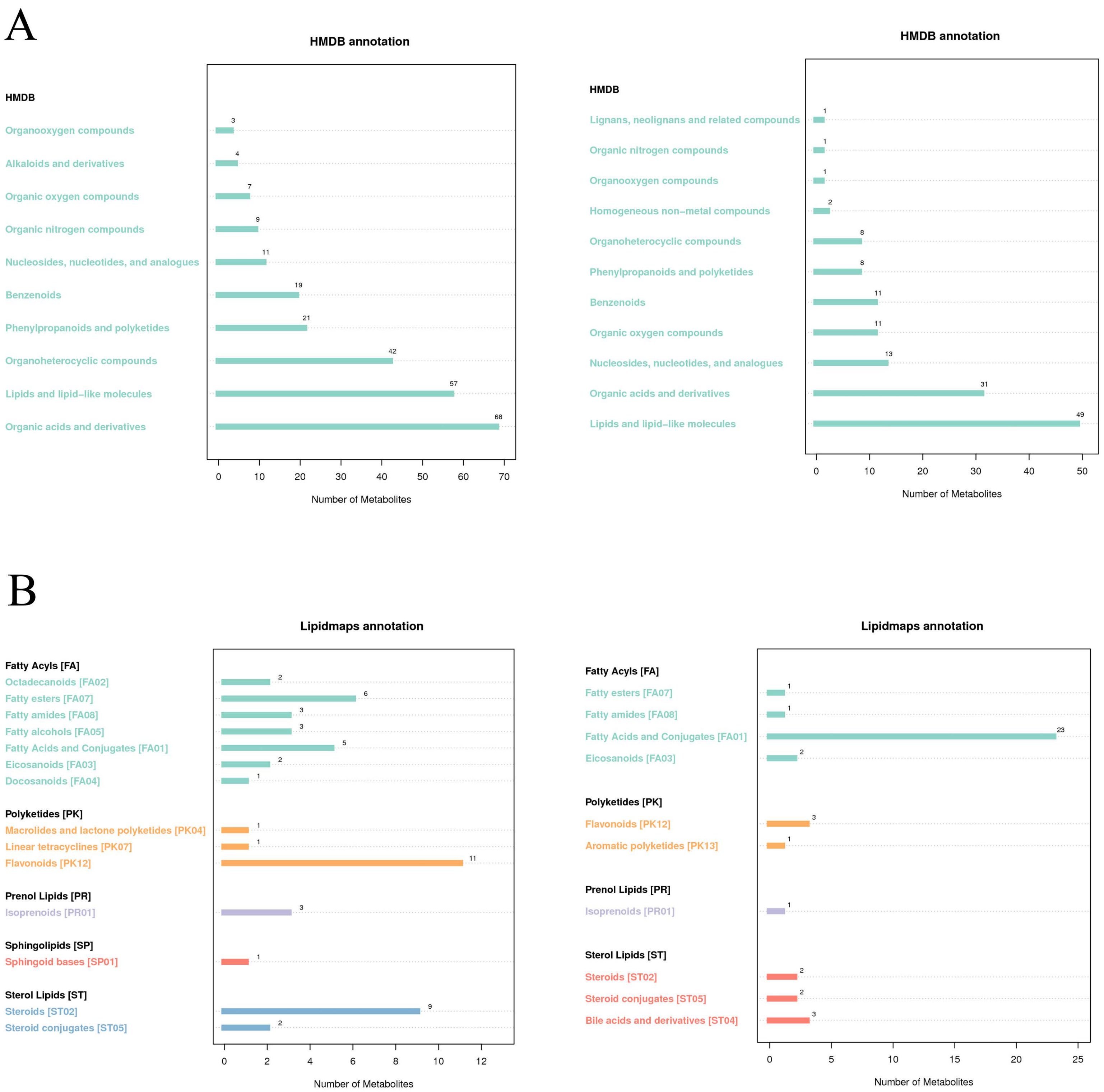
A
HMDB annotation
HMDB
Organooxygen compounds 3
Alkaloids and derivatives 4
Organic oxygen compounds 7
Organic nitrogen compounds 9
Nucleosides, nucleotides, and analogues 11
Benzenoids 19
Phenylpropanoids and polyketides 21
Organoheterocyclic compounds 42
Lipids and lipid–like molecules 57
Organic acids and derivatives 68
0 10 20 30 40 50 60 70
Number of Metabolites
HMDB annotation
HMDB
Lignans, neolignans and related compounds 1
Organic nitrogen compounds 1
Organooxygen compounds 1
Homogeneous non–metal compounds 2
Organoheterocyclic compounds 8
Phenylpropanoids and polyketides 8
Benzenoids 11
Organic oxygen compounds 11
Nucleosides, nucleotides, and analogues 13
Organic acids and derivatives 31
Lipids and lipid–like molecules 49
0 10 20 30 40 50
Number of Metabolites
B
Lipidmaps annotation
Fatty Acyls [FA]
Octadecanoids [FA02] 2
Fatty esters [FA07] 6
Fatty amides [FA08] 3
Fatty alcohols [FA05] 3
Fatty Acids and Conjugates [FA01] 5
Eicosanoids [FA03] 2
Docosanoids [FA04] 1
Polyketides [PK]
Macrolides and lactone polyketides [PK04] 1
Linear tetracyclines [PK07] 1
Flavonoids [PK12] 11
Prenol Lipids [PR]
Isoprenoids [PR01] 3
Sphingolipids [SP]
Sphingoid bases [SP01] 1
Sterol Lipids [ST]
Steroids [ST02] 9
Steroid conjugates [ST05] 2
0 2 4 6 8 10 12
Number of Metabolites
Lipidmaps annotation
Fatty Acyls [FA]
Fatty esters [FA07] 1
Fatty amides [FA08] 1
Fatty Acids and Conjugates [FA01] 23
Eicosanoids [FA03] 2
Polyketides [PK]
Flavonoids [PK12] 3
Aromatic polyketides [PK13] 1
Prenol Lipids [PR]
Isoprenoids [PR01] 1
Sterol Lipids [ST]
Steroids [ST02] 2
Steroid conjugates [ST05] 2
Bile acids and derivatives [ST04] 3
0 5 10 15 20 25
Number of Metabolites
