# Supplementary figures and images for: Effects of Herbal Tea Residue on Growth Performance, Meat Quality, Muscle Metabolome, and Rumen Microbiota Characteristics in Finishing Steers
Source: Front Microbiol. 2022 Jan 18;12:821293. doi: 10.3389/fmicb.2021.821293 (PMC8804378; doi:10.3389/fmicb.2021.821293)

class CN RE

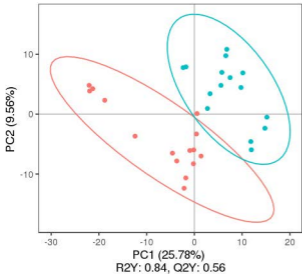

class CN RE

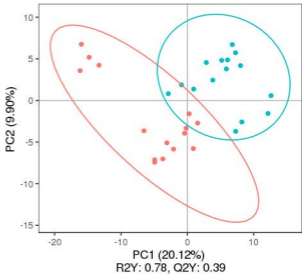

Supplement: Supplementary Figure 2 — The PLS-DA scatter plots of each sample in positive (left) and negative (right) modes. The abscissa is the score of the sample on the first principal component; The ordinate is the score of the sample on the second principal component; R2Y represents the explanatory rate of the model, and Q2Y is used to evaluate the predictive ability of the PLS-DA model. When R2Y is greater than Q2Y, the model is well established. [file Image_2.pdf]

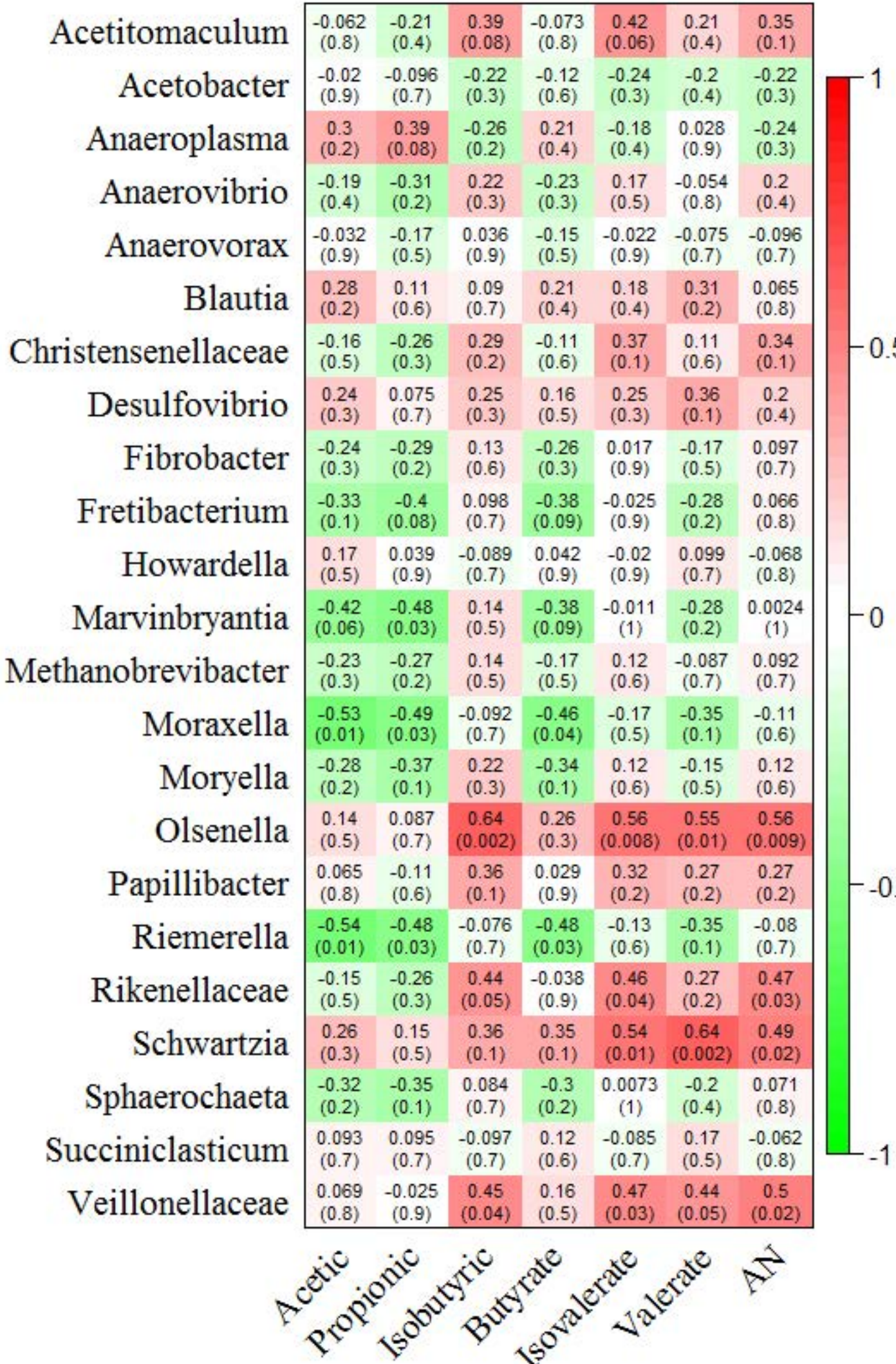

Supplement: Supplementary Figure 3 — Correlation analysis of rumen microorganisms with rumen volatile fatty acid concentrations. Each cell contains the corresponding correlation and P-value. The table is color-coded by correlation according to the color legend. [file Image_3.pdf]
