# Supplementary material for: Effects of Herbal Tea Residue on Growth Performance, Meat Quality, Muscle Metabolome, and Rumen Microbiota Characteristics in Finishing Steers
Source: Front Microbiol. 2022 Jan 18;12:821293. doi: 10.3389/fmicb.2021.821293 (PMC8804378; doi:10.3389/fmicb.2021.821293)
Supplement: Supplementary file 4 [file Table_1.DOCX]

**Table S1 Nutrient composition of herbal tea residue (HTR)**

| **components** | **As sampled basis** | **Dry matter basis** |
| --- | --- | --- |
| Moisture (%) | 75.10 |  |
| Dry matter (%) | 24.90 |  |
| Crude protein (%) | 3.26 | 13.10 |
| Crude fat (%) | 0.65 | 2.60 |
| Ash (%) | 1.67 | 6.69 |
| Acid detergent fiber (ADF, %) | 9.91 | 39.80 |
| Neutral detergent fiber (NDF, %) | 13.52 | 54.30 |
