# Supplementary material for: Effects of Herbal Tea Residue on Growth Performance, Meat Quality, Muscle Metabolome, and Rumen Microbiota Characteristics in Finishing Steers
Source: Front Microbiol. 2022 Jan 18;12:821293. doi: 10.3389/fmicb.2021.821293 (PMC8804378; doi:10.3389/fmicb.2021.821293)
Supplement: Supplementary file 5 [file Table_2.DOCX]

**Table S2** **Effects of herbal tea residue feed on amino acid contents of beef (g/100g)**

| **Items** | **CN** | **RE** | ***P* value** |
| --- | --- | --- | --- |
| Asp | 7.37±0.128 | 7.27±0.125 | 0.591 |
| Thr | 3.64±0.063 | 3.60±0.077 | 0.608 |
| Ser | 2.91±0.050 | 2.91±0.047 | 0.954 |
| Glu | 12.23±0.196 | 11.96±0.237 | 0.391 |
| Gly | 3.89±0.239 | 3.91±0.321 | 0.972 |
| Ala | 4.77±0.073 | 4.72±0.102 | 0.671 |
| Cys | 0.72±0.070 | 0.78±0.074 | 0.553 |
| Val | 3.97±0.067 | 3.90±0.074 | 0.431 |
| Met | 2.15±0.057 | 2.06±0.076 | 0.372 |
| IIe | 3.73±0.067 | 3.65±0.081 | 0.435 |
| Leu | 6.54±0.119 | 6.49±0.125 | 0.772 |
| Tyr | 2.73±0.062 | 2.64±0.059 | 0.290 |
| Phe | 3.24±0.063 | 3.18±0.057 | 0.492 |
| Lys | 7.29±0.127 | 7.19±0.152 | 0.620 |
| His | 3.37±0.105 | 3.25±0.083 | 0.368 |
| Arg | 5.25±0.069 | 5.19±0.099 | 0.648 |
| Pro | 3.40±0.117 | 3.90±0.173 | 0.985 |

Note: The values were calculated as the means ± standard error of the mean (N=15). P < 0.05 indicated significant difference between the two groups, P >0.05 indicated no significant difference between the two groups. CN, no herbal tea residues; RE, 50% HTRs replaced *Pennisetum purpureum*.
